# 1-MT inhibits the invasion of CBP-resistant ovarian cancer cells via down-regulating IDO expression and re-activating immune cells function

**DOI:** 10.1186/s40360-020-00439-w

**Published:** 2020-09-11

**Authors:** Huihan Ma, Qian Qin, Jiaqing Mi, Qinmei Feng

**Affiliations:** 1 Shanxi Medical University, No. 29 East Shuangta Street, Taiyuan, 030012 Shanxi China; 2 Department of Gynecology Medicine, People’s Hospital Affiliated to Shanxi Medical University, No. 56 Xinjian Nan Lu street, Yingze District, Taiyuan, 030000 Shanxi Province China

**Keywords:** Ovarian cancer, Indoleamine 2, 3-dioxygenase (IDO), 1-methyl-tryptophan (1-MT), Chemotherapy resistant

## Abstract

**Background:**

The indoleamine 2, 3-dioxygenase (IDO) inhibitor 1-methyl-tryptophan (1-MT) is currently being used in clinical trials in patients with relapsed or refractory solid tumors by inhibiting tumor immune escape. A greater understanding of IDO activity is required to begin to understand the molecular mechanism by which drugs work. This study was conducted to investigate of the clinical significance of 1-methyl-tryptophan (1-MT) in treating carboplatin-resistant (CBP-resistant) ovarian cancer and its mechanism of action.

**Methods:**

Using a medium dose, intermittent treatment method, a clinically relevant CBP-resistant human ovarian cancer cell line (SKOV3/CBP) was established. SKOV3/CBP cells were treated with normal serum (control) or 1-MT (0.25 ng/mL) for 4 h (SKOV3/CBP + 1-MT). Cell proliferation, invasion and IDO expression in SKOV3, SKOV3/CBP and SKOV3/CBP + 1-MT cells were determined by MTT assays, Matrigel invasion chambers assays and ELISAs, respectively. The half-maximal inhibitory concentration (IC_50_) and resistance index (RI) were also calculated. The killing ability of the NK cells and CD8+ T cells co-cultured with SKOV3, SKOV3/CBP and SKOV3/CBP + 1-MT cells were determined by LDH activity assays and the INF-γcounting method.

**Results:**

The SKOV3/CBP cell line displayed an increased IC_50_ compared to the SKOV3 cell line (*P* < 0.05) under CBP treatment. Treatment with 1-MT significantly decreased the IC_50_ and RI of SKOV3/CBP cells. Furthermore, 1-MT treatment not only reduced the invasion ability, but also suppressed IDO expression in the drug-resistant SKOV3/CBP + 1-MT cell line as compared to the SKOV3/CBP cell line. Furthermore, 1-MT enhanced the killing ability of NK cells and the amount of INF-γsecreted from CD8+ T cells which were co-cultured with the SKOV3/CBP cell line.

**Conclusion:**

Our data suggested that 1-MT inhibits the invasion of CBP-resistant ovarian cancer cells via down-regulation of IDO expression which leads to re-activation of immune cell function. We provide a conceptual foundation for the clinical development of 1-MT as an anti-tumor immunomodulator for chemotherapy resistant ovarian cancer patients.

## Background

Ovarian cancer is one of the common tumors in the female reproductive organs, with the first most common cause of cancer mortality among gynecological malignant tumors worldwide [1]. Although cytoreductive surgery and platinum-based chemotherapy remain the gold standard treatments, the 5-year overall survival rates of ovarian cancer patients remain low, in part, because of the development of drug resistance [2, 3]. Therefore, novel immunotherapeutic strategies are urgently needed to further improve the survival of chemotherapy resistant ovarian cancer patients.

Indoleamine 2,3-dioxygenase (IDO) is an immunosuppressive enzyme which is detected in many human tumors [4–6]. IDO induces immunosuppression by allowing tumor to cells to escape T lymphocytes based on regulation the content of tryptophan in tumor microenvironment through tryptophan metabolism pathway in vitro and in vivo evidence, suggesting IDO inhibitors may be efficacious novel immunotherapy compounds [7, 8]. Recently, clinical trials combining chemotherapy and IDO inhibitors, such as 1-methy-D-tryptophan (1-MT) and NLG919, for treatment of human tumors have commenced [9–12]. Such approaches have not been attempted in ovarian tumors and the mechanism by which IDO regulates tumor progression in this setting is unknown.

This study investigates of the role of the IDO inhibitor (1-MT) in treating carboplatin-resistant (CBP-resistant) ovarian cancer. We aimed to clarify the relationship between IDO expression and ovarian cancer progression, and to develop an IDO-targeted molecular therapy to inhibit the progression of ovarian cancer.

## Methods

### Cell line and reagents

The human serous cystadenocarcinoma ovarian cancer cell line SKOV3 (BNCC310551) was purchased from the Shanghai cell bank (Shanghai, China). MTT cytotoxic kit was purchased from Wuhan BOSTER Biological Technology Co., LTD (Wuhan, Hubei Province, China). Indoleamine 2,3 dioxygenase kit was purchased from the Elabscience Biotechnology Co., LTD (Wuhan, Hubei Province, China). Carboplatin was purchased from Qilu pharmaceutical Co., LTD (Jinan, Shandong Province, China). Matrigel matrix adhesive was purchased from BD company of America (Franklin Lake, New Jersey, USA). Lactate dehydrogenase (LDH) assay kit was purchased from Nanjing Bioengineering Institute (Nanjing, Jiangsu Province, China). The CD8+ T cell separation kit was purchased from STEMCELL Company (Beijing, China) and the ELISPOT kit of CD8+ T cells was purchased from RD Company (Minnesota, USA). Human peripheral blood was collected from the group of experimental healthy volunteers.

### Ethical approval and consent to participate

This study was reviewed and approved by the Ethical Committee of Shanxi Provincial People’s Hospital before extracting peripheral blood of the healthy human participants. The participants were recruited from August 2018 to December 2018. All of the participants have a unique identification number. All experiments were performed in accordance with relevant guidelines and regulations. Informed consent was obtained from study participants according to institutional guidelines.

### CBP-resistant cell line induction

The SKOV3 cell line was cultured in McCoy’s 5A medium supplemented with 10% fetal bovine serum and 10% penicillin-streptomycin mixture. When cell density reached 70–80%, carboplatin (10 μg/mL) was added to the cell culture for 1 h and then culture medium was replaced with normal medium. 50% of cells died after 24 h. When the remaining cells reached a density of 70–80%, the carboplatin treatment was repeated. This repeat treatment cycle was performed 2–6 times. For each subsequent treatment, the carboplatin concentration was increased by 50% until there was no cell death after drug treatment. The carboplatin resistant cell line, SKOV3/CBP, is stable when cultured in the presence of 70 μg/mL carboplatin.

### IDO expression analysis

The cell lines (SKOV3, SKOV3/CBP and SKOV3/CBP + 1-MT) were cultured in six well plates and the culture medium was replaced after the cells fully adhered. Cells were collected by removing culture medium and adding 0.25% trypsin. 10^5^ cells were centrifuged and washed three times with PBS. After repeated freeze-thaw cycles to lyse cells, the cell lysate was centrifugated at 20000 x g for 10 min to remove debris. The IDO expression of each sample was detected by enzyme-linked immunoassay.

### Drug resistant analysis

Drug resistant analysis was performed using an MTT assay. Cells including SKOV3, SKOV3/CBP and 1-MT (0.25 ng/mL) treatment, SKOV3/CBP cell SKOV3/CBP + 1-MT, were trypsinized and gently dissociated into a single cell suspension. 10^5^ cells were seeded into each well of a 96-well culture plate and incubated at 37 °C and 5% CO_2_. Once cells adhered the culture medium was replaced. A gradient concentration of carboplatin was added in culture medium. After 48 h of culture, 20 μl MTT solution (5 mg/mL) was added to each well and the cells were cultured in the incubator for a further 4 h. The supernatant fluid was removed and 200 μl dimethyl sulfoxide was added to each well. The absorbance (A) value of each well was detected at a wavelength of 490 nm using a spectrophotometer. Each experiment was performed in triplicate. The resistance index (RI) was calculated as follows:

SKOV3/CBP resistance index (RI) = IC_50_ (SKOV3/CBP) / IC_50_ (SKOV3), SKOV3/CBP + 1-MT resistance index (RI) = IC_50_ (SKOV3/CBP + 1-MT) / IC_50_ (SKOV3).

### Cell proliferation analysis

Cells were cultured as above. The absorbance value of the cells was detected using the MTT method after 12, 24, 48, 72 and 96 h. Using these data, a cell proliferation curve was plotted.

### Cell invasion assay

An aliquot of 100 μl Matrigel extra-cellular matrix and 100 μl serum-free medium was mixed. The Matrigel matrix solution was evenly distributed in a prechilled transwell chamber. The chamber was incubated at 37 °C overnight. 100 μl of each cell suspension (SKOV3, SKOV3/CBP and 1-MT SKOV3/CBP + 1-MT cell lines) was added to the upper chamber of a transwell chamber supplemented with the serum-free medium. 500 μl 20% fetal calf serum was added to the lower chamber. The chamber was cultured in an incubator (37 °C and 5% CO_2_) for 48 h. Cells above the Matrigel matrix were carefully removed. The remaining cells were fixed in 4% polyformaldehyde for 20 min. The values for invasion were obtained by counting 6 fields per membrane.

### Detecting the killing ability of NK cells

An equal volume of PBS was added to healthy human peripheral blood containing an anticoagulant. The resultant mixture of human anticoagulant peripheral blood was added in equal volume, to a lymphocyte separation fluid. It was then centrifugated at 1200 x g for 20 min. The lymphocyte cells were collected and washed three times with PBS. The concentration of peripheral blood lymphocytes was adjusted to 1 × 10^6^/mL. Meanwhile, SKOV3, SKOV3/CBP and SKOV3/CBP + 1-MT cells were collected by 0.25% trypsinization. Peripheral blood lymphocytes (effector cells) were mixed at a ratio of 5:1, 10:1, 20:1 and 40:1 with SKOV3, SKOV3/CBP and SKOV3/CBP + 1-MT cells (target cells) in a 96-well plate. After 48 h of co-culture, the supernatant was collected and the LDH activity was determined using an LDH activity assay kit. LDH activity was calculated as follows: LDH activity (U/L) = (treatment OD value - control OD value) / (standard OD value - blank OD) × standard concentration (0.2 μmol/L) × 1000.

### CD8+ T cell mediated cytotoxicity assay

Anticoagulant heparin solution was added to whole human blood and incubated for 20 min. Subsequently, an equal volume of PBS + 2% FBS was added each blood sample. The mixture was added to an equal volume of lymphocyte separation solution and centrifuged at 1200 x g for 20 min at room temperature. CD8+ T cells were separated and washed three times with PBS + 2% FBS. The purity of CD8+ T cells was greater than 90% when measured by flow cytometry.

The concentration of CD8+ T cells (effector cells) was adjusted to 1 × 10^6^/mL in RPMI-1640 medium. Meanwhile the SKOV3, SKOV3/CBP and SKOV3/CBP + 1-MT cells (target cells) were trypsinized and resuspended to a concentration of 1 × 10^4^/mL. An aliquot of 100 μl cells (effector cells: target cells = 100:1) was added to each well in a 96-well plate which contained a flat nitrocellulose membrane pre-coated with INF-γ antibody. Two control wells, with negative blank control and a positive ConA control (ConA 10 μg/mL) were used. The cells were removed from the plate after 24 h and cultured in an incubator (37 °C and 5% CO_2_). Culture medium was removed and 200 μl deionized subaqueous cell lysis was added to each well. The culture plate was washed with washing buffer. The washed culture plate was incubated with a biotin-labeled antibody for 1 h. After that, avidin was added to the washed culture plate. Finally, after a 3-amino-9-ethylcarbazole stain, deionized water was added to terminate the staining process and the culture plates were dried at room temperature for 30 min. The spot number of the 96-well plate was measured by an ELISPOT counting instrument. The spot number /1 × 10^6^ CTL was quantified and the frequency of INF-γ secretion from CD8+ T cells was calculated.

### Statistical analysis

SPSS 11.5 software was used for statistical analysis. Normally distributed data was analyzed using a one-way ANOVA. Data is shown as mean ± standard deviation. *P* < 0.05 is considered as statistically significant.

## Results

### IDO expression analysis

**Fig. 1:**
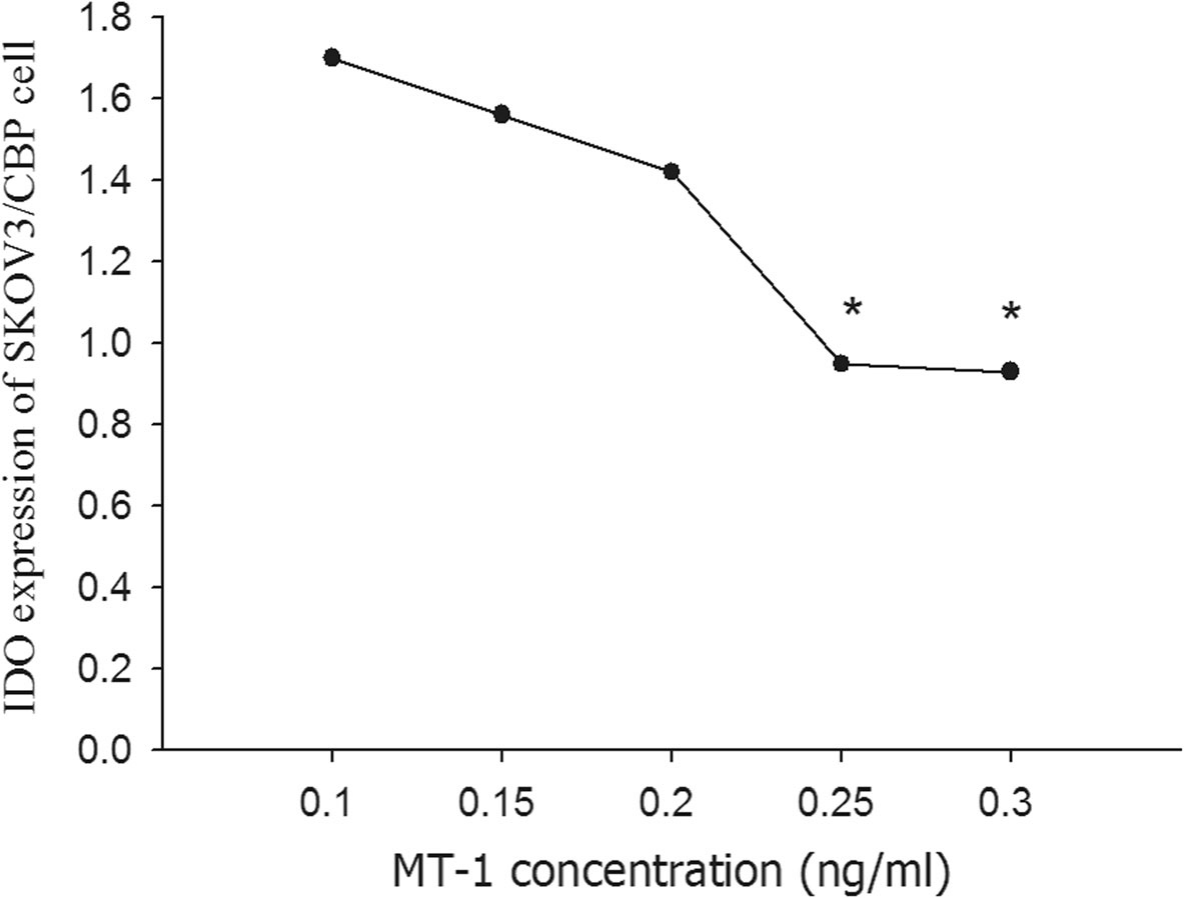
IDO expression in SKOV3, SKOV3/CBP and SKOV3/CBP + 1-MT cell lines. Note:*P < 0.05 represents SKOV3/CBP VS SKOV3, **P < 0.05 represents SKOV3/CBP + 1-MT VS SKOV3/CBP

The expression of IDO in response to a range of 1-MT concentrations was examined (Fig. 1). With increasing 1-MT concentration the expression of IDO decreased gradually. The inhibitory effect of 1-MT plateaued at 0.25 ng/mL, therefore, the concentration of 1-MT used in subsequent experiments was 0.25 ng/mL.

**Fig. 2:**
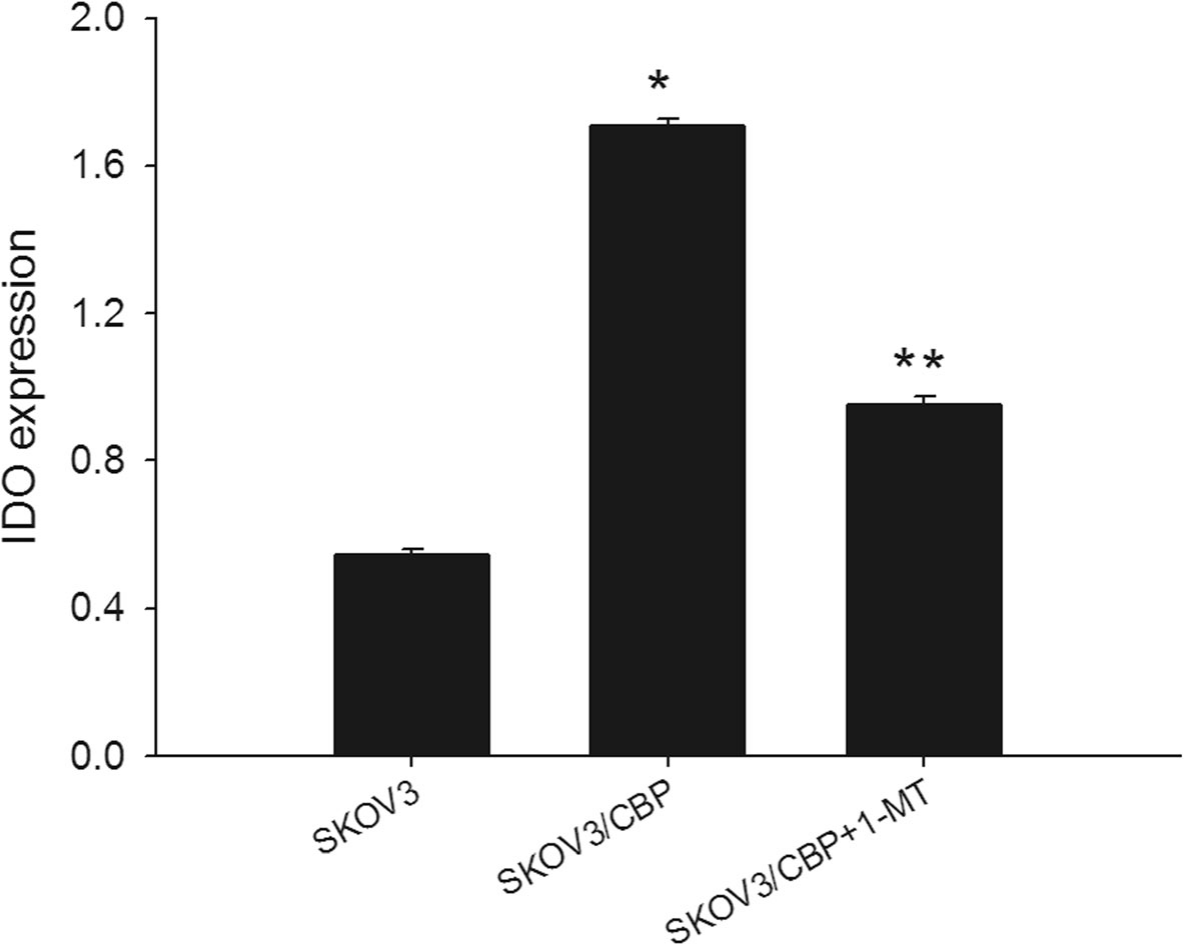
IDO expression in the SKOV3/CBP cell line at a range of 1-MT. concentrations

The results showed that IDO was expressed in the SKOV3, SKOV3/CBP and SKOV3/CBP + 1-MT cell lines. IDO expression in the drug-resistant SKOV3/CBP cell line was significantly higher than SKOV3 cells and IDO expression was significantly decreased in CBP-resistant SKOV3 cell after 1-MT treatment (Fig. 2). These results suggest that IDO expression is increased in drug-resistant ovarian cancer cells. 1-MT treatment partially decreases IDO expression in SKOV3/CBP cells, although it doesn’t fully deplete IDO expression to the endogenous levels observed in SKOV3 cells.

### Half-maximal inhibitory concentration and resistance index (RI) analysis

The CBP-resistant ovarian cancer cell line (SKOV3/CBP), which was developed by using a moderate-dose and intermittent treatment method, grows stably in the presence of 70 μg/mL carboplatin. Half-maximal inhibitory concentration (IC_50_) and resistance index (RI) in SKOV3, SKOV3/CBP and SKOV3/CBP + 1-MT cell lines were calculated with a linear fit. The IC_50_ of the SKOV3/CBP cell line was significantly higher than the SKOV3 cell line (Table 1). The IC_50_ and RI of SKOV3/CBP + 1-MT cells was significantly lower than the SKOV3/CBP cell line, suggesting that 1-MT treatment decreases the survival rate and drug resistance of the CBP-resistant cells.

**Table 1:** IC_50_ and RI of the ovarian cancer cells under carboplatin treatment

Cell line	IC50 (ug/mL)	RI
SKOV3	23.1	
SKOV3/CBP	90.9^a^	3.93
SKOV3/CBP + MT-1	63.5^b^	2.75^b^

Note: ^a^ represent SKOV3/CBP VS SKOV3, ^b^ represent SKOV3/CBP + MT-1 VS SKOV3/CBP *P*<0.05. IC50: Half maximal inhibitory concentration; RI: resistance index

### Cell proliferation analysis

**Fig. 3:**
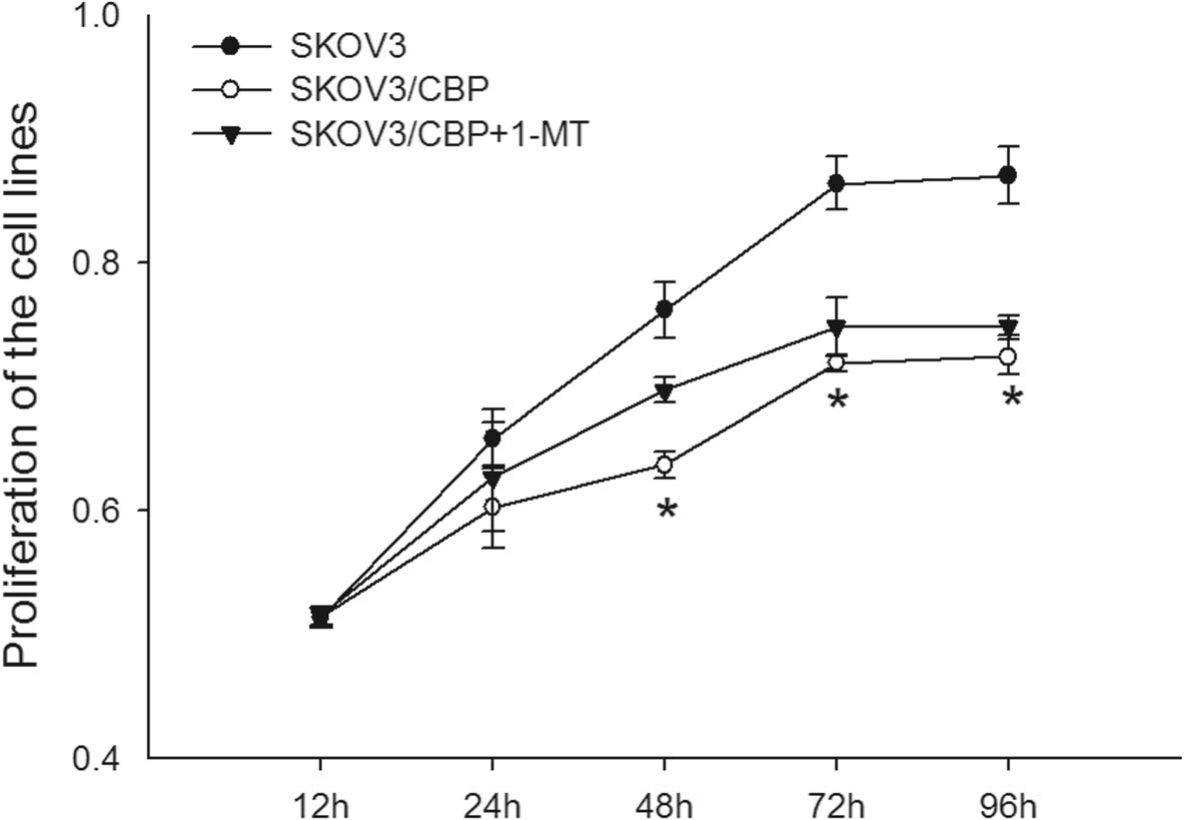
Proliferation of the SKOV3, SKOV3/CBP and SKOV3/CBP + 1-MT cell lines. Note: *P < 0.05 represents SKOV3/CBP VS SKOV3, **P < 0.05 represents SKOV3/CBP + 1-MT VS SKOV3/CBP

The proliferation rates of SKOV3, SKOV3/CBP and SKOV3/CBP + 1-MT cell lines are shown in Fig. 3. Proliferation of the SKOV3/CBP cell line was significantly lower than the SKOV3 cell line after 48 h. Proliferation of the SKOV3/CBP + 1-MT cell line was higher than the SKOV3/CBP cell line. There was no significant difference in the cell proliferation between the SKOV3/CBP cell line and SKOV3/CBP + 1-MT cells, indicating that 1-MT treatment had no significant effect on SKOV3/CBP proliferation.

### Invasion ability analysis

**Fig. 4:**
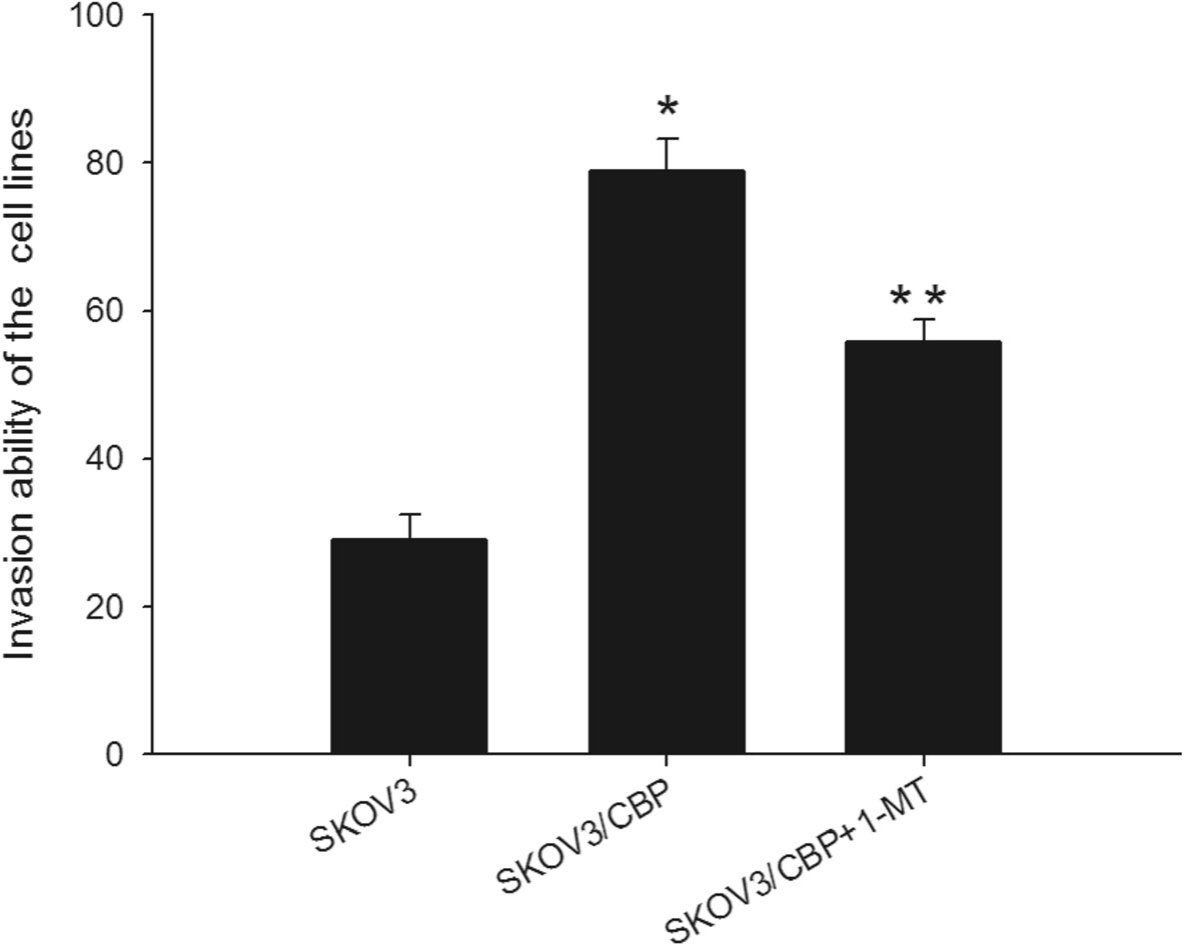
Invasion ability of the SKOV3, SKOV3/CBP and SKOV3/CBP + 1-MT cell lines. Note:*P < 0.05 represents SKOV3/CBP VS SKOV3, **P < 0.05 represents SKOV3/CBP + 1-MT VS SKOV3/CBP

The invasion ability of the CBP-resistant SKOV3/CBP cell line was significantly higher than the SKOV3 cell line (Fig. 4). Cell invasion ability was significantly decreased in response to 1-MT treatment in the SKOV3/CBP cell line. This suggests that 1-MT inhibits the invasion ability of the SKOV3/CBP cell line and may be associated with CBP-resistant cell recurrence and metastasis.

### Killing ability of NK cells co-cultured with SKOV3/CBP cells

**Fig. 5:**
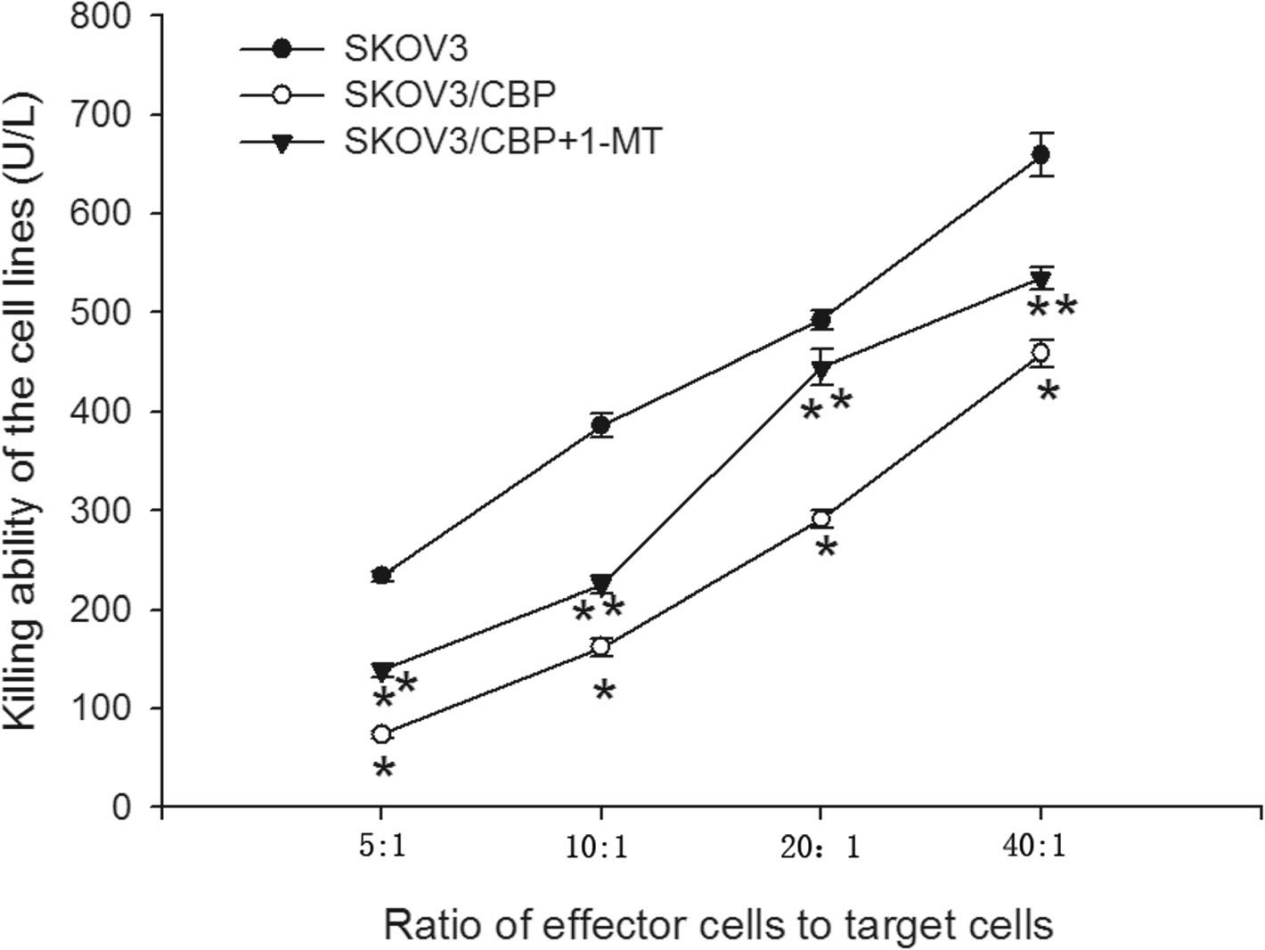
Killing ability of the NK cells which were co-cultured with SKOV3, SKOV3/CBP and SKOV3/CBP + 1-MT cell lines as measured by LDH activity assay. Note:*P < 0.05 represents SKOV3/CBP VS SKOV3, **P < 0.05 represents SKOV3/CBP + 1-MT VS SKOV3/CBP

The killing ability of NK cells which were co-cultured with SKOV3, SKOV3/CBP and SKOV3/CBP + 1-MT cells was detected using an LDH assay. With an increased ratio of effector cells to target cells, the killing ability of NK cells was increased (Fig. 5). The killing ability of NK cells when co-cultured with SKOV3/CBP cells was significantly decreased as compared to when co-cultured with SKOV3 cells. Interestingly, the killing ability of NK cells when co-cultured with SKOV3/CBP + 1-MT cells was significantly higher than when co-cultured with SKOV3/CBP cells, suggesting that 1-MT increases the killing ability of NK cells.

### INF-γ secretion in CD8+ T cells co-cultured with SKOV3/CBP cells

**Fig. 6:**
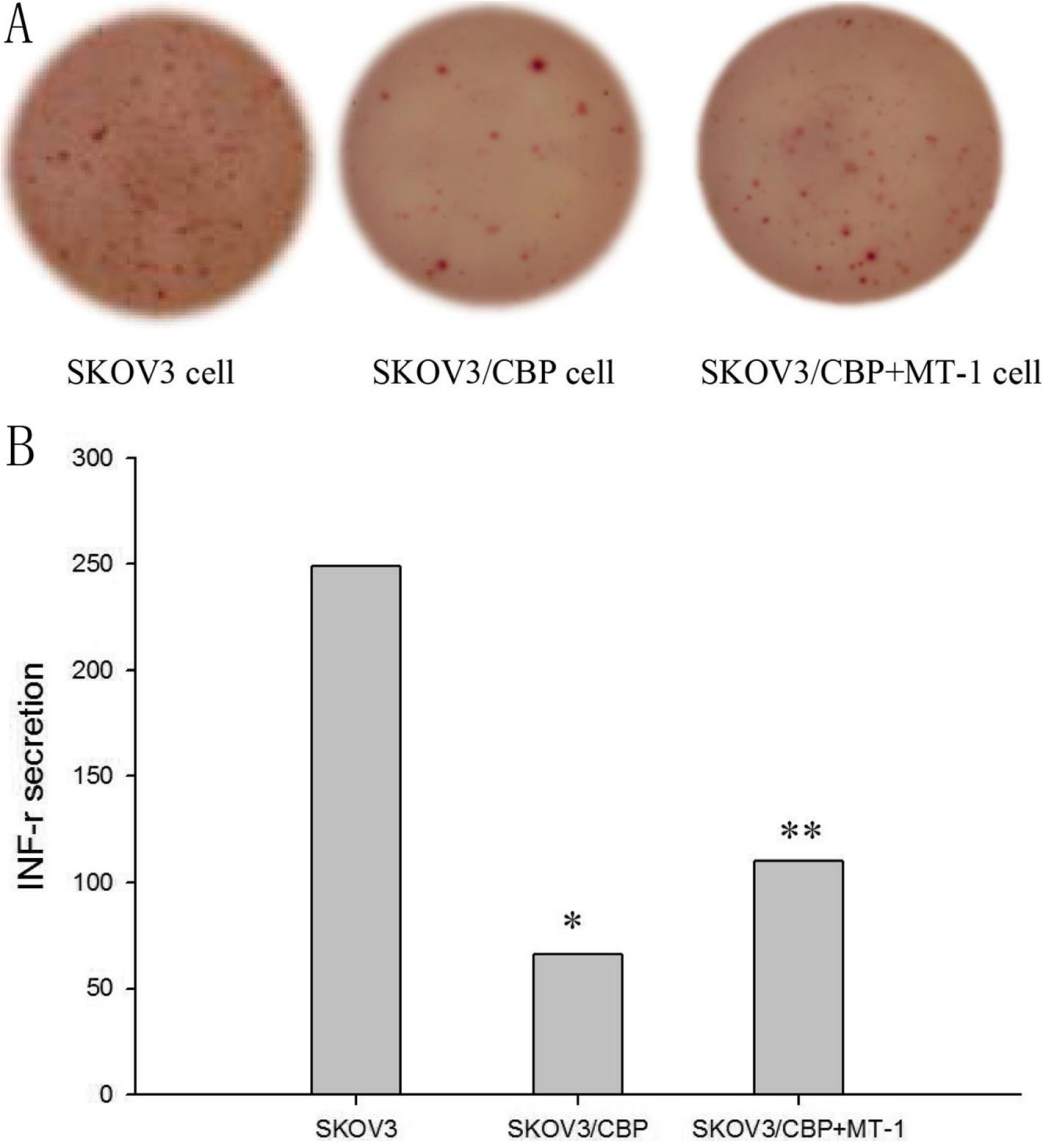
INF-γ secretion from CD8+ T cells which were co-cultured with SKOV3, SKOV3/CBP or SKOV3/CBP + 1-MT cell lines. Note: *P < 0.05 represents SKOV3/CBP VS SKOV3, **P < 0.05 represents SKOV3/CBP + 1-MT VS SKOV3/CBP

The amount of INF-γ secreted from CD8+ T cells which were co-cultured with SKOV3/CBP cells was significantly decreased as compared to when co-cultured with SKOV3 cells (Fig. 6). This indicates that the killing ability of CD8+ T cells is decreased in the presence of drug-resistant ovarian cancer cells. The amount of INF-γ secreted from CD8+ T cells which were co-cultured with SKOV3/CBP cells was significantly increased upon 1-MT treatment (*P*<0.05), indicating that 1-MT treatment increases the sensitivity of lymphocytes and killing ability of CD8+ T cells.

## Discussion

Our research aimed to clarify the relationship between the immunosuppressive enzyme IDO and CBP-resistant ovarian cancer cells. The CBP-resistant ovarian cell line, SKOV3/CBP, was created to examine the relationship between the IDO and CBP resistance in vitro. In addition, we investigated the effect of the IDO inhibitor, 1-MT, on CBP-resistant ovarian cancer cells and clinically relevant immune cells in vitro.

It has been demonstrated that IDO expression positively correlates with chemoresistance in paclitaxel-based chemotherapy in ovarian cancer [13]. IDO expression is observed in several gynecological tumors, such as ovarian cancer, cervical cancer, endometrial cancer and breast cancer [4, 14, 15]. As expected, IDO expression was detected in the SKOV3, SKOV3/CBP and SKOV3/CBP + 1-MT cell lines used in this study. It has been reported that IDO expression is decreased in toremifene resistant patients compared to non-resistant patients [16]. Conversely, in our experiments, it was demonstrated that IDO expression is increased in the CBP-resistant SKOV3/CBP cell line as compared to the non-resistant cell line. These converse findings may be a result of the different chemotherapeutic drugs used or the in vivo in vitro differences. The IDO inhibitor, 1-MT, significantly decreased IDO expression in SKOV3/CBP cells. These results demonstrate that the expression of IDO is associated with CBP-resistance in ovarian cancer cells.

In this study, we found that the proliferation rate of the SKOV3/CBP cell line was significantly lower than that of the SKOV3 cell line, whereas the invasion ability of the SKOV3/CBP cell line was significantly higher than that of the SKOV3 cell line. This suggests that the drug-resistance mechanism of the SKOV3/CBP cells may be increased invasion ability rather than increased proliferative potential. Further investigation showed that 1-MT treatment did not enhance proliferation of SKOV3/CBP cells in vitro, but significantly suppressed invasion ability. These findings indicate that IDO is associated with CBP-resistance in ovarian cancer cells by altering the invasion ability of drug-resistant cells, not the proliferation ability.

IDO expression is thought to play a role in an endogenous feedback mechanism which controls excessive immune responses [4, 17]. It has been reported that IDO induces the accumulation of the tryptophan metabolite kynurenine, which inhibits NK cell and T cell function and results in an immunosuppressive state in the tumor microenvironment [18, 19]. Tumor cells continue to develop and metastasize by using this negative immune regulation to evade identification and killing by the immune system [20]. In our study, the killing ability of NK cells and INF-γsecretion from CD8+ T cells co-cultured with SKOV3/CBP cells was decreased as compared to co-culture with SKOV3 cells. 1-MT treatment partly reinstated the killing ability of NK cells and INF-γsecretion from CD8+ T cells co-cultured with SKOV3/CBP cells in vitro. Similarity, Della Chiesa et al. reported that IDO induces the accumulation of the tryptophan metabolite kynurenine, which suppresses NK cell receptor expression, thereby inhibiting NK cell function [21]. Uttenhove et al. reported that, in IDO-expressing tumors, IDO promotes local tryptophan degradation and depletion, resulting in suppressed T-cell function and local immunotolerance [4]. Thus, it appears that IDO expression and the suppression of NK/T cell function are involved in the increased invasion ability of CBP-resistant ovarian cancer.

Recent studies in animal models suggest that 1-MT combined with paclitaxel and cyclophosphamide chemotherapy drugs significantly inhibit tumor growth. Further research showed that 1-MT effectively slows the growth of tumor cells and enhances the effect of chemotherapy drugs through significantly enhancing the sensitivity of the immune system to tumor antigens and enhancing the expression of IL-2 levels within the tumor microenvironment [22–24]. These results show that 1-MT can reduce the expression of IDO and reverse carboplatin resistance in ovarian cancer cells. Furthermore, it can increase the killing ability of immune cells and thus reduce the infiltration or metastasis of tumors. In addition, IDO inhibition represents an alternative immunotherapeutic strategy to overcome the immunosuppressive tumor microenvironment in anti-PD1-resistant tumors [25]. Therefore, IDO inhibitors will provide a new therapeutic strategy for ovarian cancer patients.

## Conclusion

Taken together, this study demonstrates that 1-MT inhibits the invasion of CBP-resistant ovarian cancer cells via down-regulation of IDO expression and re-activation of immune cell function. This suggests that the IDO inhibitor, 1-MT, is a potentially effective immunotherapy for ovarian cancer.

## Data Availability

The datasets used and analyzed in the current study are available from the corresponding author upon request.

## Abbreviations

IDO: Indoleamine 2,3-dioxygenase
1-MT: 1-methyl-tryptophan
CBP-resistant: carboplatin-resistant
IC_50_: Half-maximal inhibitory concentration
RI: resistance index
